# Radiographic Determination of Hip Rotation Center and Femoral Offset in Japanese Adults: A Preliminary Investigation toward the Preoperative Implications in Total Hip Arthroplasty

**DOI:** 10.1155/2015/610763

**Published:** 2015-10-21

**Authors:** Taichiro Takamatsu, Takaaki Shishido, Yasuhito Takahashi, Toshinori Masaoka, Toshiyuki Tateiwa, Kosuke Kubo, Kenji Endo, Masaya Aoki, Kengo Yamamoto

**Affiliations:** ^1^Department of Orthopaedic Surgery, Tokyo Medical University, 6-7-1 Nishishinjuku, Shinjuku-ku, Tokyo 160-0023, Japan; ^2^Department of Bone and Joint Biomaterial Research, Tokyo Medical University, 6-7-1 Nishishinjuku, Shinjuku-ku, Tokyo 160-0023, Japan

## Abstract

The values of hip rotation center (HRC) and femoral offset (FO) evaluated according to Caucasian anatomical landmarks have been regarded as a useful reference also for Japanese patients in total hip arthroplasty (THA). In a strict sense, however, since there can be racial differences among their anatomical morphologies, it is clinically important to reconsider those parameters for the Japanese. In the present study, in order to investigate correlations among hip and pelvic morphometric parameters, frontal radiographs were taken from 98 Japanese adults (60 males and 38 females) without acetabular dysplasia and arthropathy in the standing position. Their mean age was 62.0 ± 16.7 years. The horizontal position of HRC was significantly correlated with the pelvic width in both genders (*P* = 0.0026 and 0.0010 for the males and the females, resp.). The vertical position of HRC was significantly correlated with the teardrop-sacroiliac distance in the males (*P* = 0.0003) and with the pelvic cavity height in the females (*P* = 0.0067). However, in both genders, there were no correlations among FO and the other parameters analyzed in this study. Our present findings might contribute to theoretical implications of an appropriate HRC position for Japanese OA patients in THA.

## 1. Introduction

Osteoarthritis (OA) of hip can be divided into primary and secondary forms [1–5]. The primary OA is generally regarded to be associated with underlying abnormalities of articular cartilage without an anatomical abnormality or specific disease process, which is more common in Caucasians than in non-Caucasians [2, 3]. On the other hand, the primary OA is a quite rare condition in Japan, and its prevalence was reported by only 0.65 ~ 9% among Japanese patients with hip OA [3–5]. Since the secondary OA involves congenital or developmental anatomic abnormalities such as acetabular dysplasia, it is particularly difficult to identify an original anatomical position for patients in total hip arthroplasty (THA). The hip rotation center (HRC) and femoral offset (FO) are important factors affecting clinical outcomes for THA, the optimization of which can lead to maximizing an implant durability and mechanical function in a replaced hip joint. In general, high positioning of an acetabular component can induce the reduced muscle strength and the increased prosthetic wear after THA, ultimately leading to the revision surgery [6–11]. When patients with the small FO stand on one leg, large strength of abductor muscle is required, and this condition increases stresses in hip prosthetic surfaces. In addition, these patients may frequently undergo the femoropelvic impingement and subsequent dislocation [12, 13].

Despite such importance for considering the anatomical parameters regarding HRC and FO, there is difficulty in precisely determining their optimal positions especially in patients who have a bilateral OA with substantial destruction of both hips (namely, due to no clear landmark available in the unaffected side of the hips). Thus, it is of great importance to rationalize the averaged target values of HRC and FO. Several regression formulae to assess HRC from pelvic morphologies have been reported previously for Caucasians [14–17]. Nevertheless, it should be noted that hip and pelvic morphologies can significantly differ by races [18–21]. In this context, it is questionable whether those formulae are directly applicable for non-Caucasians or not. To date, there have been few studies on rationalizing HRC and FO values for Japanese OA patients. In this study, we evaluated HRC and FO by using the plain frontal radiographs of the hips (including the pelvises) for the ultimate goal of determining their appropriate values for Japanese adults.

## 2. Methods

### 2.1. Subjects and the Studied Morphometric Parameters

This study was preliminary approved by the Institutional Review Board (IRB) of Tokyo Medical University, and a written informed consent was provided before undergoing any study-related procedures. We performed prospective study in a total of 98 Japanese adults (60 males and 38 females) hospitalized continuously at our institution for an orthopaedic disorder elsewhere except OA. Note that we did not include the patients who had arthropathy as well as acetabular dysplasia with a sharp angle of >45° and CE angle of <20°. In addition, the patients with joint space narrowing and spur formation were not included in this analysis. The mean age of those patients was 62 years (ranging from 16 to 90 years), and we obtained frontal radiographs of the hips (including the pelvises) for the assessment of HRC and FO in the standing position as previously performed in the similar studies [14–16, 22]. The morphological evaluations using the radiographs were made in both hips of each patient, and then the obtained values were averaged.

**Figure 1:**
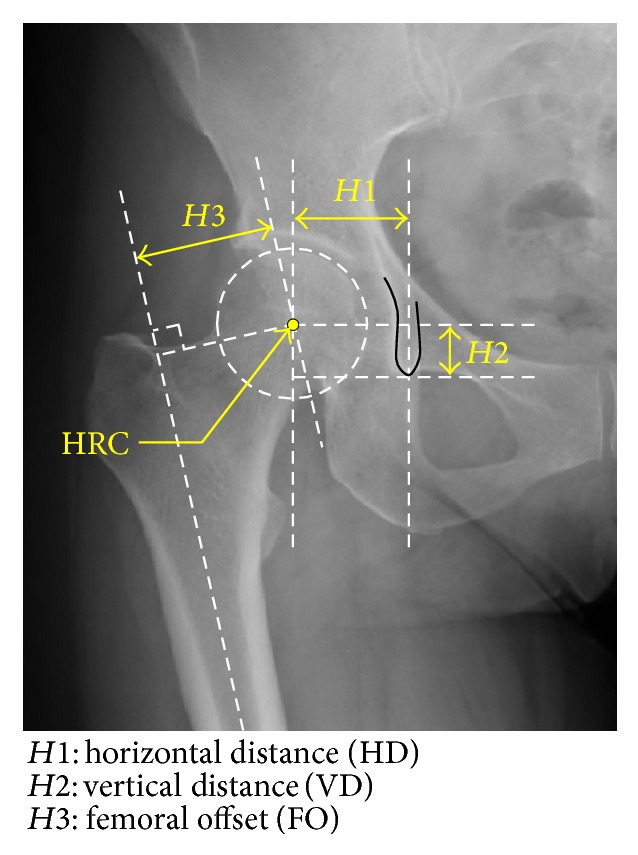
Hip morphometric parameters (*H*1–*H*3) analyzed in this study (cf.  Section 2.1).

The evaluated hip morphometric parameters of* H*1–*H*3 were shown in Figure 1, the definitions of which were given as follows:

*H*1: horizontal distance (HD, length in perpendicular line between HRC and vertical line across inferior edge of teardrop center).
*H*2: vertical distance (VD, length in perpendicular line between HRC and tangential line to inferior edge of teardrop center).
*H*3: femoral offset (FO, length in perpendicular line between HRC and femoral axis).

**Figure 2:**
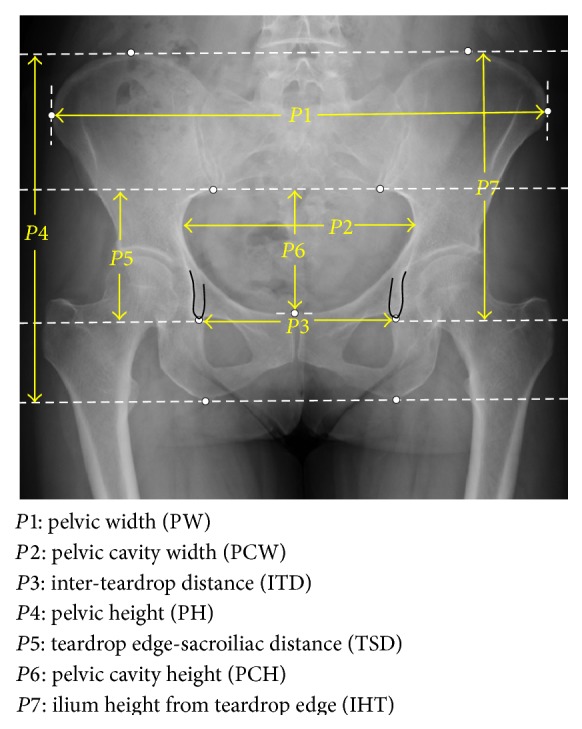
Pelvic morphometric parameters (*P*1–*P*7) analyzed in this study (cf.  Section 2.1).

In addition, we evaluated the pelvic morphometric parameters of* P*1–*P*7 shown in Figure 2, the definitions of which were given as follows:

*P*1: pelvic width (PW).
*P*2: pelvic cavity width (PCW).
*P*3: interteardrop distance (ITD).
*P*4: pelvic height (PH).
*P*5: teardrop edge-sacroiliac distance (TSD).
*P*6: pelvic cavity height (PCH).
*P*7: ilium height from teardrop edge (IHT).

All these parameters were assessed by several observers (at least three orthopaedic surgeons among the authors of this study). The measured values were averaged and were eventually recorded as the analyzed parameters of* H*1–*H*3 and* P*1–*P*7.

### 2.2. Statistical Analysis

Quantitative variables were described by the mean ± SD. Pearson's correlation coefficients (*r*) were calculated and used to evaluate bivariate linear relations among the studied parameters. The variability of sensitivity and specificity estimates was indicated by 95% exact confidence intervals. A software package SPSS version 20.0 (SPSS Inc., Chicago, IL, USA) was used for the statistical analysis. A *P* < 0.05 was considered statistically significant.

## 3. Results

**Figure 3:**
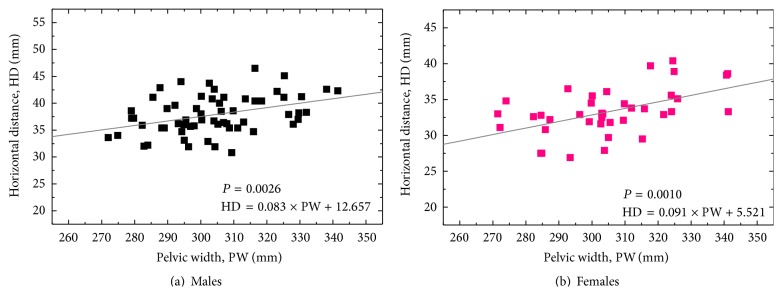
Correlation between pelvic width (PW) and horizontal distance (HD, length in perpendicular line between HRC and vertical line across inferior edge of teardrop center) in the Japanese males (a) and females (b). A linear line of fit is given in each diagram (*r* = 0.381, *n* = 60, *P* = 0.0026, and HD = 0.083 × PW + 12.657, for the males; *r* = 0.51, *n* = 38, *P* = 0.0010, and HD = 0.091 × PW + 5.521, for the females).

**Figure 4:**
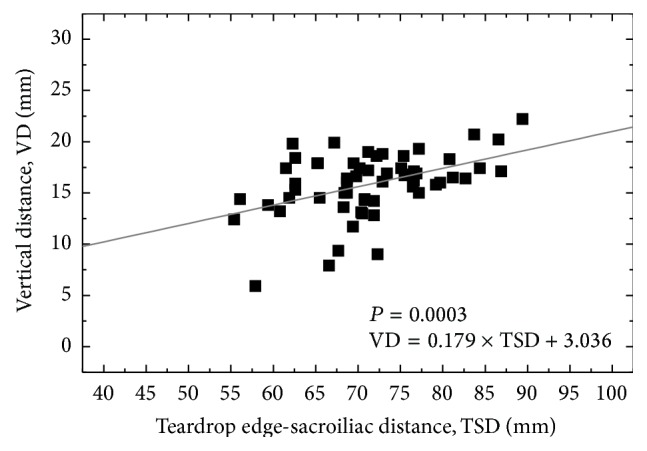
Correlation between teardrop edge-sacroiliac distance (TSD) and vertical distance (VD, length in perpendicular line between HRC and tangential line to inferior edge of teardrop center) in the Japanese males. A linear line of fit is given in the diagram (*r* = 0.45, *n* = 60, *P* = 0.0003, and VD = 0.179 × TSD + 3.036).

**Figure 5:**
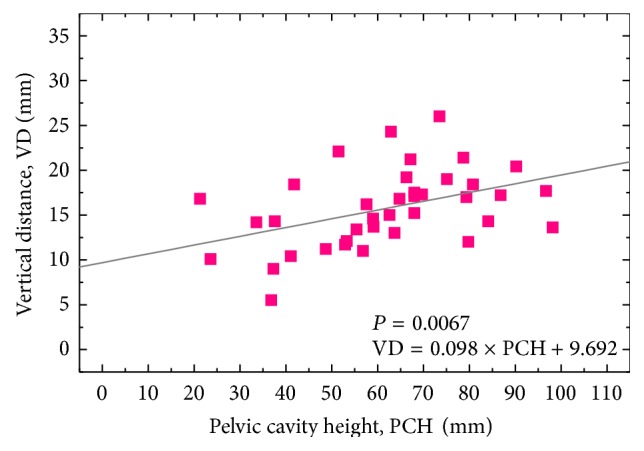
Correlation between pelvic cavity height (PCH) and vertical distance (VD, length in perpendicular line between HRC and tangential line to inferior edge of teardrop center) in the Japanese females. A linear line of fit is given in the diagram (*r* = 0.43, *n* = 38, *P* = 0.0067, and VD = 0.098 × PCH + 9.692).

The mean values of HD, VD, and FO for the Japanese males were obtained as 37.8 ± 3.5, 15.8 ± 3.0, and 36.0 ± 5.8 mm, while those for the females were 33.3 ± 3.2, 15.7 ± 4.2, and 33.4 ± 4.9 mm, respectively (Table 1). Figures 3(a)-3(b) show the relationship between HD and PW in the males and females, respectively. As depicted in Figure 3, significant positive correlations were noted between HD and PW values in both genders (*r* = 0.381 and *P* = 0.0026 for the males, and *r* = 0.51 and *P* = 0.0010 for the females). The solid lines are a liner regression fit which indicates the relationship as follows: HD = 0.083 × PW + 12.657 (for the males) and HD = 0.091 × PW + 5.521 (for the females). In the Japanese males, a significant positive correlation was also observed between VD and TSD values (*r* = 0.45, *P* = 0.0003, and VD = 0.179 × TSD + 3.036) (Figure 4). On the other hand, in the Japanese females, a significant positive correlation was found between VD and PCH values (*r* = 0.43, *P* = 0.0067, and VD = 0.098 × PCH + 9.692) (Figure 5). However, in both genders, FO parameter was not correlated with any other morphological parameters analyzed in this study.

## 4. Discussion

The geometrical effects of HRC and FO on a clinical outcome of THA have been previously discussed by several authors [6–17]. The mathematical considerations made by Johnston et al. [6] suggested that the improved mechanical function can be accomplished by placing HRC medially, inferiorly, and anteriorly. In other words, HRC reconstructed at high and lateral position can increase resultant forces and moments generated in prosthesis, possibly inducing a formation of wear debris and subsequent implant loosening [6–11]. On the other hand, it was reported that a lowering of FO reduces an abductor muscle force, moment arm, and range of motion, which can impose an increased risk of impingement between greater trochanter and acetabulum wall [14–17]. Nevertheless, an excessive FO can result in the increased stresses to femoral neck and also in pain at greater trochanter area [23].

Although, in principle, the position of HRC in OA patients should be reconstructed at the original position of their acetabula at the time of THA, there were indeed no rigorous anatomical landmarks for Japanese patients. The unaffected contralateral hip can provide a good anatomical indication for HRC for individual patient, but, as mentioned in the Introduction section, it is quite difficult to determine an appropriate HRC value especially in the case of patient affecting the bilateral hip OA.

To date, various methodologies and regression formulae for determining HRC have been reported via radiographic analyses. Ranawat et al. [14] suggested constructing an isosceles triangle with side lengths of 0.2 × PH from the point located at 5 mm lateral to the intersection of Koeler's and Shenton's lines. Pierchon et al. [15] reported that HD = 0.2 × ITD in males and HD = 0.18 × ITD in females, and HD = 0.3 × PCH in males and HD = 0.25 × PCH in females. Fessy et al. [16] reported that VD = 0.204 × TSD − 0.794 in both genders. John and Fisher [17] suggest placing HRC at the position of 0.13 × PH lateral and 0.07 × PH superior to the teardrop in both genders. In addition, Schofer et al. [22] compared the methodological validations among them and concluded that the calculation according to Fessy et al. [16] provided the most precise and reliable determination of HRC (i.e., VD = 0.204 × TSD − 0.794). On the other hand, in the present study analyzed in Japanese population, the regression formulae were obtained as HD = 0.083 × PW + 12.657 and VD = 0.179 × TSD + 3.036 (for the males) and HD = 0.091 × PW + 5.521 and VD = 0.098 × PCH + 9.692 (for the females). The only similarity between Fessy's equation and the present ones can be found in the positive correlations between VD and TSD in the males (cf. Figure 4), but the slope of the regression formula is much lower in our study than Fessy's formula (0.098 versus 0.204). In the Japanese females, VD did not significantly correlate TSD, but PCH. The above differences in the regression formulae can be interpreted due to racial and gender variations in their hip and pelvic morphologies [18–21]. Lavy et al. [21] reported that Japanese hips were more dysplastic than British hips and that, within the same racial group, women were more dysplastic than men. Such differences in the intrinsic hip morphology, thus, could lead to the variations of HD, VD, and FO values depending on races as well as genders.

A pioneering study on HRC analyzed in Japanese population was conducted by Fujii et al. [24], which showed that the males had the hip parameters of HD = 39.0 ± 3.0 and VD = 15.0 ± 3.2 mm and the females had those of HD = 34.1 ± 2.7 and 15.4 ± 2.9 mm. These values show excellent agreement with those obtained in the present study (i.e., HD = 37.8 ± 3.5 and VD = 15.8 ± 3.0 for men; HD = 33.3 ± 3.2 and VD = 15.7 ± 4.2 for women). On the other hand, we obtained the mean FO value as 36.0 ± 5.8 and 33.4 ± 4.9 mm for the Japanese men and women, respectively, and a similar FO value was previously reported as 37.1 ± 4.6 mm in Japanese population [25]. However, the mean FO values in Western populations were markedly different from those in the Japanese, which were reported as 43.0 ± 6.8 [26], 42.6 (ranging from 26.9 to 53.9) [27], and 42.2 ± 5.1 mm [28]. The current results obtained from the Japanese showed that the FO value was not significantly correlated with any pelvic morphometric parameters.

Finally, we mention that this study had the following limitations. The unmatched numbers in the subjects between men and women were examined (*n* = 60 for men, and *n* = 38 for women) and the study involved their broad age ranges (62.0 ± 16.7 years). In addition, since the conventional plane radiographs correspond to the projection on the frontal view in a 2-dimensional (2D) plane, the FO measurement might lead to providing relatively larger errors or variations depending on rotation conditions of the femurs (cf. femoral anteversion and external rotation) as compared to the other studied parameters. Sariali et al. [28] found that the 3-dimensional (3D) morphological data analyzed by using computed tomography (CT) scanning provided 3.5 mm larger FO in white patients undergoing THA, as compared to 2D measurement of its projection on the frontal plane. Therefore, 3D CT analysis would be more preferable to find anatomical correlations regarding FO, but it must rely on the future study. Despite the above limitations, the present radiographic analyses showed positive correlations between HRC and pelvic morphometric parameters in Japanese adults (HD versus PW and VD versus TSD, for the males; HD versus PW and VD versus PCH for the females), which might be conducive to appropriately defining the proper hip anatomic landmarks.

By most experienced hip surgeons, it can be (theoretically) agreed that the optimum locations for HRC and FO of THA are the original anatomical positions, but, from the practical viewpoints of hip reconstructions, the theoretical placement of prostheses would not be always applicable due to the presence of large acetabular defects. In such difficult cases, the reconstructions can be performed by standard-sized cemented cups with bone impaction grafting [29, 30], custom-made prostheses [31], oversized prostheses [32, 33], and high HRC (typically, in the order of several millimeters superior to the anatomical center) [8, 34]. Russotti and Harris [8] explained that high HRC in THA was not a crucial parameter for long-term fixation of cemented acetabular components. In addition, they recommended placing an acetabular cup at a more superior but not more lateral position of HRC in a difficult acetabular reconstruction [8]. In addition, it was also reported that superior cup position was not associated with concomitant lateralization [8, 34]. However, it was confirmed that high HRC can result in significantly higher aseptic loosening rates in femur rather than acetabulum [7, 34] as well as the increased risk of dislocation from impingement [34]. In the above contexts, it has not been fully clarified yet how much nonanatomical positioning of HRC can be allowed in terms of the long-term stability in the fixation. Therefore, it is of great importance to more rigorously define the “safe zone” of HRC and FO for THA, and we believe that the present results will contribute to promoting such studies in the future.

## Figures and Tables

**Table 1:** Mean values and standard deviation (mean ± SD, mm) of hip morphometric parameters analyzed in the Japanese males and females.

	Males	Females	Total
Horizontal distance, HD (mm)	37.8 ± 3.5	33.3 ± 3.2	36.2 ± 4.0
Vertical distance, VD (mm)	15.8 ± 3.0	15.7 ± 4.2	15.2 ± 3.8
Femoral offset, FO (mm)	36.0 ± 5.8	33.4 ± 4.9	34.3 ± 5.8
